# Supplementary material for: The Effects of Oral Semaglutide on Hepatic Fibrosis in Subjects with Type 2 Diabetes in Real-World Clinical Practice: A Post Hoc Analysis of the Sapporo-Oral SEMA Study
Source: Pharmaceuticals (Basel). 2025 Jan 19;18(1):129. doi: 10.3390/ph18010129 (PMC11769496; doi:10.3390/ph18010129)
Supplement: Supplementary file 1 [file pharmaceuticals-18-00129-s001.zip › Supplementary Table_02_20250110_kitsunai_Creared.pdf]

Table S1. Breakdown of subjects in whom changes in the FIB-4 index could not be measured.

Missing data	N	
(-) Fib-4 index (baseline and 6 months)		2
(-) Age	1	
(-) Platelet	1	
(-) Fib-4 index (baseline)		1
(-) Platelet	1	
(-) Fib-4 index (6 months)		12
(-) AST, (-) ALT, (-) Platelet	2	
(-) AST	2	
(-) ALT	6	
(-) Platelet	2	
**Total**		15

AST, aspartate aminotransferase; ALT, alanine aminotransferase

Table S2. Relationship between the changes in indices for liver steatosis/fibrosis pre- and post-treatment with oral-semaglutide and baseline subjects' characteristics.

**Variables**	**Changes in HSI**		**Changes in FIB-4 index**	
	**ϱ**	***P*-values**	**ϱ**	***P*-values**
Age	−0.026	0.738	−0.115	0.138
Body mass index	0.149	0.055	−0.102	0.190
HbA1c	−0.039	0.616	−0.058	0.459
AST	0.178	0.021	−0.489	< 0.001
ALT	0.092	0.241	−0.235	0.002
γ-GTP	0.288	< 0.001	−0.338	< 0.001
eGFR	0.006	0.938	0.008	0.913
HSI	0.035	0.659	0.085	0.271
FIB-4 index	0.129	0.097	−0.368	< 0.001

Data were analyzed using Spearman's rank-correlation. HbA1c, glycated hemoglobin; AST, aspartate aminotransferase; ALT, alanine aminotransferase; γ-GTP, γ-glutamyl transpeptidase; eGFR, estimated glomerular filtration rate; HSI, hepatic steatotic index.

Table S3. Changes in the indices of liver steatosis and fibrosis in subjects aged ≥65 years at high risk for liver fibrosis (FIB-4 index ≥2.0).

Variables	Hepatic steatosis index			FIB-4 index		
	**Baseline**	**6 months**	***P*-values**	**Baseline**	**6 months**	***P*-values**
Total (*n* = 15)	39.0 ± 4.9	39.2 ± 5.5	0.823	2.69 (2.19-3.07)	2.07 (1.30-2.81)	0.030

Data are shown as the mean ± SD or median (25%–75%). *P*-values were obtained using the paired *t*-test or Wilcoxon test.
