# Supplementary figures and images for: The Effects of Oral Semaglutide on Hepatic Fibrosis in Subjects with Type 2 Diabetes in Real-World Clinical Practice: A Post Hoc Analysis of the Sapporo-Oral SEMA Study
Source: Pharmaceuticals (Basel). 2025 Jan 19;18(1):129. doi: 10.3390/ph18010129 (PMC11769496; doi:10.3390/ph18010129)

Figure S1 \*  $p < 0.05$ , \*\*  $p < 0.01$ , and \*\*\*  $p < 0.001$

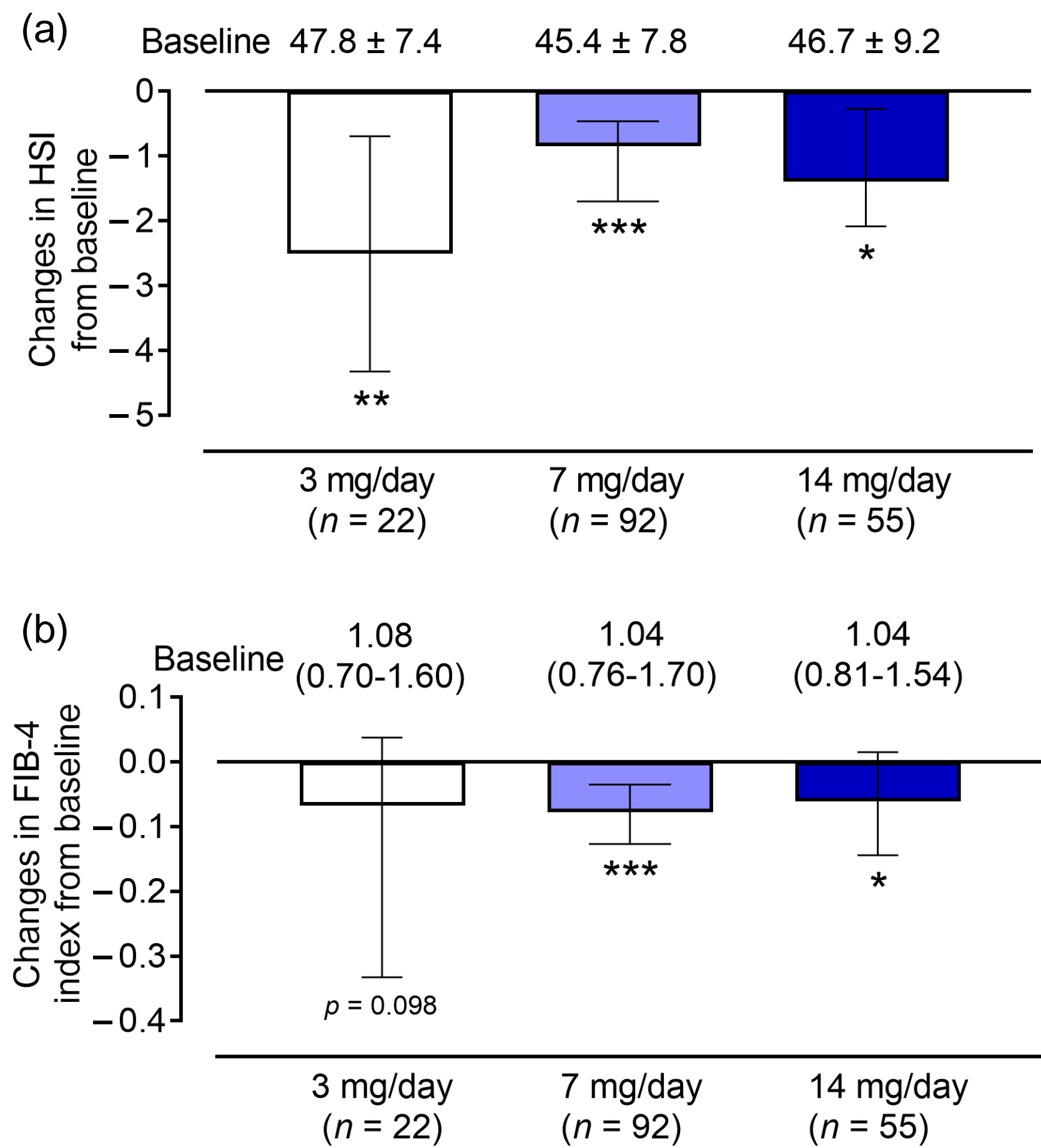

Supplement: Supplementary file 1 [file pharmaceuticals-18-00129-s001.zip › Supplementary Figure S1 pharmaceuticals-3412452.pdf]
